# Competing interests: digital health and indigenous data sovereignty

**DOI:** 10.1038/s41746-024-01171-z

**Published:** 2024-07-04

**Authors:** Ashley Cordes, Marieke Bak, Mataroria Lyndon, Maui Hudson, Amelia Fiske, Leo Anthony Celi, Stuart McLennan

**Affiliations:** 1 Environmental Studies Program and Department of Data Science, University of Oregon, Eugene, OR USA; 2 Institute of History and Ethics in Medicine, Department of Preclinical Medicine, TUM School of Medicine and Health, Technical University of Munich, Munich, Germany; 3 Department of Ethics, Law and Humanities, Amsterdam UMC, University of Amsterdam, Amsterdam, The Netherlands; 4 Centre for Medical and Health Sciences Education, School of Medicine, The University of Auckland, Auckland, New Zealand; 5 Te Kotahi Research Institute, University of Waikato, Hamilton, New Zealand; 6 Division of Pulmonary, Critical Care, and Sleep Medicine, Beth Israel Deaconess Medical Center, Boston, MA USA; 7 Laboratory for Computational Physiology, Massachusetts Institute of Technology, Cambridge, MA USA; 8 Department of Biostatistics, Harvard T.H. Chan School of Public Health, Boston, MA USA; 9 Institute for Biomedical Ethics, University of Basel, Basel, Switzerland

**Keywords:** Health policy, Medical research

## Abstract

Digital health is increasingly promoting open health data. Although this open approach promises a number of benefits, it also leads to tensions with Indigenous data sovereignty movements led by Indigenous peoples around the world who are asserting control over the use of health data as a part of self-determination. Digital health has a role in improving access to services and delivering improved health outcomes for Indigenous communities. However, we argue that in order to be effective and ethical, it is essential that the field engages more with Indigenous peoples´ rights and interests. We discuss challenges and possible improvements for data acquisition, management, analysis, and integration as they pertain to the health of Indigenous communities around the world.

## Introduction

There are ~476 million Indigenous people around the world, belonging to Indigenous communities spread across >90 countries with different languages, worldviews, relations to land, preferred terminology and most relevant: determinants of health^1^. Many Indigenous groups share a commonality of inclusion under the United Nations Declaration of Rights of Indigenous people, but even within national contexts Indigenous peoples are far from a cultural or political monolith, with 574 different Indigenous Nations represented within the United States alone^2,3^. Existing research overwhelmingly indicates that health outcomes are generally poorer in Indigenous communities^4^. These inequities are heightened in pandemics, as COVID-19 has once again demonstrated, with some Indigenous communities experiencing significantly higher infection and death rates and lower vaccination rates^5^. Digital health has a role in improving access to services and delivering improved health outcomes for Indigenous communities. However, we argue that in order to be effective, it is essential that the field engages more with Indigenous peoples´ rights and interests.

The field of digital health is increasingly promoting open health^6–9^. Although this open approach to data promises a number of benefits, it also leads to tensions with Indigenous data sovereignty movements led by Indigenous peoples around the world who are asserting control over the use of health data as a part of self-determination. For some Indigenous communities, the premise of open access as “good for all” is fundamentally flawed and digital technologies have the potential to put Indigenous traditional knowledge and customary practices at risk of global appropriation^10^. Further, mistrust exists more broadly between many Indigenous communities and dominant societies, due to differing histories of colonialism, genocide, and historic exclusions^11^. Given these complex dynamics, data sovereignty is a critical component of Indigenous peoples’ inherent, political, and digital sovereignties, which must constantly be re-asserted due to ongoing power asymmetries. At the same time, Indigenous peoples acknowledge the potential for digital technologies that rely upon more ethically collected data to promote Indigenous health and flourishing^12^. In this Perspective, we discuss that tension and potential steps forward, after providing some background on the concept of Indigenous data sovereignty.

## Indigenous data sovereignty

For Indigenous communities, data can be a strategic ‘resource’ to inform policies and improve outcomes^13^. Within an Indigenous rights framework, communities have asserted data sovereignty and instituted governance over their data, which are forms and records of Indigenous knowledge^14,15^. Indigenous data sovereignty is defined as “the right of Indigenous Peoples to own, control, access and possess data that derive from them, and which pertain to their members, knowledge system, customs or territories” (p. 654)^16^. Acknowledging the tension between protecting Indigenous rights and supporting open data, the International Indigenous Data Sovereignty Interest Group published the ‘CARE Principles for Indigenous Data Governance’ (Collective Benefit, Authority to Control, Responsibility, and Ethics)^17^. The CARE principles have the primary goals of “fostering Indigenous self-determination by enhancing Indigenous use of data for Indigenous pursuits” (p. 3)^17^, and honouring the FAIR Principles (Findable, Accessible, Interoperable, Reusable) for data management and stewardship, while ensuring data sharing on Indigenous terms and being more people- and purpose-focused to complement the data centric nature of the FAIR Principles^18^. The CARE Principles respond to the increasing need for Indigenous participation in data governance activities, given that a lot of Indigenous data is stewarded within non-Indigenous institutions. The framework also draws upon existing standards created in more local contexts such as The First Nations Principles of Owners, Control, Access and Possession (OCAP) that apply to ceremonial, economic, and health data as well as other data categories^19^.

Framed around the CARE Principles, the Research Data Alliance later published the ‘COVID-19 Guidelines for Data Sharing Respecting Indigenous Data Sovereignty’^20^, to support greater inclusion of Indigenous peoples in pandemic-related research and planning. The guideline recommended that Indigenous data rights, priorities, and interests be recognised in COVID-19 research activities across the data lifecycle, and in any subsequent innovations. It also set out the requirements for Indigenous-designed data approaches and standards, inclusive of the rights to Indigenous management of data governance and decision-making within the planning and design of Indigenous data collection and sharing. The guideline also highlights the inadequacy of personal and individual data privacy protections for Indigenous peoples, and how collective data privacy protections, supported via community-controlled data infrastructure, are essential to ethical Indigenous data practices^20^.

The advancement of Indigenous data sovereignty guidelines has worked to address longstanding issues surrounding inclusion, representation, and ownership of digital health data. Nonetheless, digital health disparities persist due to remaining structural health inequalities and difficulties translating guidelines into practice. This is particularly apparent during moments of crisis or upheaval, such as when many Indigenous communities found themselves excluded from public health interventions during the COVID-19 pandemic, with agencies reverting to centralised decision-making processes for both data collection and service delivery. Indeed, frustration with the lack of attention and progress towards increasing vaccination rates in Indigenous communities led some to challenge government processes (for an example from Aotearoa/New Zealand, see: Supplementary note 1).

## Challenges for Indigenous data governance

To identify barriers and potential steps forward in upholding Indigenous data sovereignty in practice, we surveyed empirical studies about the experiences and reflections of both non-Indigenous researchers and Indigenous communities and scientists, specifically in the area of health data research. A literature search (see Text Box 1) included a total of 24 articles from the United States, Canada, Australia, Aotearoa/New Zealand, and Sweden, focused on diverse communities (Aboriginal Australians, Alaska Natives, Māori, Métis, Native Americans, Native Hawaiians, Pacific Islanders, Sámi peoples, Torres Strait Islanders). We identified challenges facing the secondary use of Indigenous health data for research with regards to the collection, use and management of data.

Box 1 Search strategy and selection criteria References were identified through a search of PubMed and Google Scholar between January 2001 and October 2023, using search terms related to (specific) Indigenous peoples and health data science (e.g., “Indigenous” or “First Nations”; “health” or “medical”; “data research”, “data sharing” or “data governance”). Other relevant references were identified through (backwards) reference list searching. Studies were included that reported empirical findings and case studies regarding barriers and/or solutions for including Indigenous peoples in health data research. Only academic papers published in English were included; ideally the literature search would have included research in Indigenous languages. Future research could also include more Indigenous centred key concepts in search criteria such as decolonisation, socio-cultural and emotional determinants of health and wellbeing, Indigenous research methodologies, and Indigenous knowledges.

### Data collection

Data of Indigenous communities is often incomplete and inaccurate, due to missing Indigenous identifiers or data aggregation that obscures local differences and often results in statistical erasures^21^. Difficulties identifying Indigenous peoples in large datasets often arise because identification is voluntary or Indigenous identifiers are not registered correctly or at all due to histories of assimilative and racist policies in many countries^22,23^. Even when Indigenous data are reported separately, the data are less meaningful when differences between local communities are not accounted for^24–30^. Although geographical data aggregation is sometimes necessary due to privacy concerns, this makes it difficult to gauge the unique health needs of individual groups^31^. Furthermore, issues of data quality often hinder the targeting of research to communities that need health services the most. For example, poor data collection of ethnicity or Indigenous affiliation limits an accurate assessment of inequalities regarding outcomes and care^32^. In some cases, data about Indigenous communities are incomplete or lacking entirely due to non-participation, research moratoriums declared by Indigenous nations, or biased recruitment^33,34^.

### Data use

Even when data are available, there is often a lack of relevant and meaningful indicators for improving Indigenous communities´ health. Indigenous models of well-being are often different from non-Indigenous models, e.g. with different ontological and epistemological understandings of spirituality, identity, relation to place, and representation in health^29,35,36^. Yet with big data analytics, studies are increasingly conducted using secondary data analysis without community participation, which further distances marginalized communities from leadership and control and produces less culturally relevant and sometimes even erroneous results^26,37,38^. The benefits of health data research are often poorly defined and indirect, and in some cases, communities do not benefit at all because study results are not used in policy decisions or support misguided decisions^29,38,39^. In other cases, insights from routinely collected data are never returned to the community and Indigenous communities need to negotiate access to their own data with research or government institutions^31^.

### Data management

Indigenous communities often lack the technological infrastructure in terms of data storage and analysis, as well as the needed logistical and fiscal structures^22,24,25,27,29,35,40,41^. Furthermore, internet infrastructure, other telecommunications, and electricity are sometimes unreliable in reservation contexts^42^. When data systems do exist, there is often a lack of interoperability with other data sources, and limited staffing capacity, as Indigenous peoples trained in data management and analysis are in high demand^25,27^^,29,35^^,41^^,43,44^. Moreover, in contrast to academic institutions, Indigenous communities and Nations do not always have equitable access to funding schemes for data science^40^.

## Towards Indigenous data sovereignty in digital health

### Improving availability, access, and accuracy

Various suggestions have been made to improve issues with Indigenous data collection. Firstly, routine self-identification of ethnicity and Indigenous identity/affiliation should be encouraged to ensure accurate registration and avoid reinforcing colonial definitions of group membership^22,25,26,28,29,31^. When this can be done securely, disaggregated data should be stored, and alternatives should be sought for counting small populations, e.g. aggregating data over multiple years instead of areas^26,41,45^. To address issues of privacy and mistrust, Indigenous identifiers may be made available to non-Indigenous researchers only after approval from a data governance committee or data can be analysed on-site by Indigenous statisticians and released as de-identified results^23,45^. The CARE principles anticipate the use of Indigenous ethical frameworks as part of data access protocols, and Ngā Tikanga Paihere is one example being used to as part of the formal approval process to access data from the Integrated Data Infrastructure in New Zealand^46^. Moreover, it is crucial that researchers are aware of the heterogeneity across Indigenous communities, which may not be appropriately captured in large-scale data collections or administrative datasets, and how the differences in cultures, histories and practices impact on their research questions^37,38^.

Secondly, examples of research with Aboriginal and Torres Strait Islander communities’ health data show that in successful digital health projects, the research is tailored to the specific community and Indigenous researchers lead the way in discussions on data collection and quality^29,43^. Similarly, in the cases of tuberculosis surveillance data from Indigenous communities in Canada, it was shown how surveillance data should be contextualized and supplemented with community-led data collection on topics like kinship, cultural healing and well-being^31^. This is found even more important in cases of stigmatized illness like hepatitis C^37^. Some authors note that if possible and productive, data should be ‘repatriated’ to Indigenous communities. For instance, Alaska Native leaders said they were more likely to approve research when data are stored within the communities^24^. However, Indigenous data sovereignty principles and requirements can also create a barrier to data sharing, for example, when they are stringent and limit collection of health data by national governments. Whenever full data repatriation would disproportionately hamper research, and in turn health outcomes among Indigenous communities, it has been suggested that partnerships should be developed where Indigenous representatives are acknowledged as rights holders who retain majority vote for decisions^30,35,47^. In those cases, a context-sensitive approach to Indigenous data sovereignty could be valuable where different types of data require different levels of “use” rights (e.g. exclusive rights for genetic data and shared rights for central government administrative data)^41^. General principles of responsible research should be accompanied by ‘living’ guideline documents and contracts related to the community concerned^26,27,30,31,35–37,40,47,48^. For instance, Love et al. describe how a legally binding Data Governance Agreement can function to create a decision space where Indigenous partners are equal participants, that is, if it includes stipulations on community returns, transparency, accountability, intellectual property and ensures co-ownership of community data and co-authorship on research publications^31^. In the case of Indigenous genomic data, some authors have suggested blockchain frameworks as a technological method for formalizing community consent and data access, that is, if the carbon footprint of these technologies can be kept to a minimum^34^.

Thirdly, for data reported at community level it is important that communities can oversee how stories about them are being created, framed, and told^47,48^. Indigenous communities may choose to review all studies or only sensitive collections, and review can be formal or informally done by Indigenous officials, governance bodies, councils, cultural boards, committee dedicated to humans research ethics, or Elders^27,48^; access by non-Indigenous researchers should be overseen by a committee populated by Indigenous peoples^23,39^. For large-scale publicly available data collections that also include Indigenous communities’ data, regulatory bodies with Indigenous representatives may need to be established by funders or researchers to oversee that protocols and data sharing agreements are in line with principles of data sovereignty such as OCAP^37,38^. Overall, there is consensus in the literature that ethics review of study protocols, transparent information provisions, community-level consent and data access are always needed, but no agreement exists on whether individual consent should be required for Indigenous health data research^22,24,31,38–41,47^. The latter is in line with unresolved debates about general digital health.

### Promoting benefits and sociocultural relevance

Issues with beneficial data use can be addressed in various ways. First, data should be used in ways relevant to Indigenous peoples, and research waste minimised. Indigenous communities themselves are taking the lead in creating relevant research agendas and measures consistent with Indigenous conceptions of wellbeing, and in cataloguing previous studies so to avoid overlap^27,28,35,37,38,40,49^. A project on hepatitis C data of First Nations communities in Ontario successfully employed a strength-based approached rather than a deficit model, by comparing outcomes within the community rather than focusing on the gap between Firs Nations and non-First Nations, and this led to better prioritization and more targeted interventions^37^. At the same time, initiatives should be set up for building cultural and historical awareness among non-Indigenous digital health researchers. The latter promotes restorative justice, trust and culturally appropriate interpretation of study results, that is, if conducted as a continuous process rather than one meeting^22,24,30,48^. Sensitivity trainings about terminology, history, inequities and Indigenous data sovereignty principles are often held on Indigenous lands with Indigenous facilitators and cultural components^23,30,37,39,44^. Second, benefits should be promoted, and harmful collaborations avoided, e.g. with companies that infringe on the territorial rights of an Indigenous Nation^44^. Benefits resulting from data science may include: obtaining actionable knowledge about the community’s health; training and employment resulting from research activities; a positive experience that may foster further appropriate data sharing and contribute to sustainable relations with non-Indigenous researchers; and free or discounted access to downstream products of the research^27,37,39,40^. Yet researchers and policy makers should be transparent about uncertainties and long timelines before realizing benefits and avoid overpromising the value of study findings^24,26,41,49^. Third, study results should be co-interpreted and evaluated with community representatives (as part of a continuous policy and planning cycle) and findings disseminated within the community before broader public dissemination^23,24,27,29,37,39^. This dissemination should be bi-directional by providing communities with options for giving feedback^38^.

### Building relations, infrastructure and Indigenous capacity

It is also apparent that addressing Indigenous data management challenges firstly requires accepting that even though Indigenous data sovereignty calls for a shift of power and control to Indigenous communities, shared responsibility and collaborative partnerships with non-Indigenous institutions remain valuable, e.g. governmental organisations may provide opportunities for centralized data collection and statistical expertise^23,26–28,33,36^. Non-Indigenous institutions should leverage their privilege to help remove barriers with Indigenous communities and should, at least initially, expect to fund most of the work^30,48^. Indigenous governance organizations (e.g. the Alberta First Nations Information Governance Centre in Canada) can also offer support by serving as ‘brokers’ between communities and researchers, or by providing free trainings on Indigenous data sovereignty as an in-kind contribution to a health data research project^31^. Clear collaboration agreements should be made through iterative negotiations and there should be plenty of room in grant budgets and timelines for (Indigenous ways of) relation building and trust-building^24,27,28,31,44,48^. Other suggestions for shaping collaborative models include hiring a community member as project coordinator^48^ or advisory board member^38^, or publishing a shared statement of values to guide the work^28,36^. In addition, the recruitment and training of Indigenous staff in data management and governance tasks, in a way that fits Indigenous worldviews^35^, is paramount for building active data leadership and promoting Indigenous data sovereignty^23,27,29,33^. In one case, for instance, recruiting two additional staff members helped reduce missing data about Native Hawaiian and Pacific Islander communities from 52 to 19%^28^.

However, there is a need to further build capacity and infrastructure for data creation, curation, analysis, and translation into practice within Indigenous communities^38^. This can be addressed through dedicated funding opportunities as well as with strategies such as hackathons and promotion of Indigenous data science curriculum. Health datathons have proved to be a powerful tool to promote collaboration between clinicians and data scientists^49^. Teams of computer scientists, engineers, nurses, pharmacists and doctors, are challenged to work together to address a clinical question or information gap over a 2–3 day event. Clinicians learn the nuances of data gathering and model development, and data scientists are provided invaluable insights into clinical data capture and decision making^49^. Courses on data sciences and health AI are another powerful tool offered and recommended in Indigenous educational institutions to create interest and capacity, drawing on ontologies and epistemologies of Indigenous peoples. Efforts such as these, organised by Indigenous communities, could promote Indigenous data sovereignty, and help build capacity for self-led data science.

## Outlook

Achieving the goals of digital health is contingent on improving disparities in care, access, and outcomes of Indigenous and other marginalised groups. In doing so, the digital health field must honour Indigenous protocols and engage with how traditional knowledge and practices of a particular nation or cultural group are inextricably tied to contemporary Indigenous data. While Indigenous voices and research permeate this Perspective, including its authorship, it approaches the topic from within a dominant paradigm of digital health. It is the responsibility of dominant and alternative paradigms, not just Indigenous peoples, to support Indigenous sovereignty, respect Indigenous protocols, and facilitate opportunities for greater participation. Moving from processes of inclusion towards co-design will also create spaces for Indigenous communities to engage in and lead actions that achieve Indigenous data sovereignty and governance in digital health. Sovereignty and community-grounded flexible control of health data and research processes remain critical for Indigenous digital health protocols. The suggestions made in this paper can provide pointers for responsible integration in specific Indigenous contexts, which will require dealing with specific legal and cultural challenges in different jurisdictions, as well as further study.

### Supplementary information

Supplementary material
