# Supplementary material for: Competing interests: digital health and indigenous data sovereignty
Source: NPJ Digit Med. 2024 Jul 4;7:178. doi: 10.1038/s41746-024-01171-z (PMC11224364; doi:10.1038/s41746-024-01171-z)
Supplement: Supplementary file 1 — Supplementary material [file 41746_2024_1171_MOESM1_ESM.pdf]

Supplementary

Supplementary note 1: Māori vaccination data during the Covid-19 pandemic

Although Aotearoa/New Zealand had one of the lowest rates of infection and mortality resulting from the COVID-19 pandemic, its COVID-19 vaccination program had not achieved equitable coverage between Indigenous Māori and other ethnic groups; the percentage of the eligible Māori population who had received COVID-19 vaccinations was materially lower than the percentage of other eligible populations. Underlying reasons for the inequity included significant barriers to Māori accessing primary healthcare services such as cost, access to services, poor service delivery, cultural barriers, poor communication by health providers, and different approaches and models to wellbeing [1]. A lack of trust by Māori in settler government institutions was also seen as a reason why the Māori vaccination rate was lower than other groups [1].

Te Tiriti o Waitangi (the Treaty of Waitangi) is Aotearoa/New Zealand's foundational document formalising the relationship between the British Crown and Māori Tribes. Established Treaty principles that are applicable to the health system in NZ include: 1) Tino Rangatiratanga – Māori self-determination over health and disability services; 2) Equity – commitment to achieving equitable health outcomes for Māori; 3) Active protection – protection of Māori health and achieving equitable health outcomes for Māori; 4) Options – provide for and properly resource Māori health and disability services; and 5) Partnership – requires the Crown and Māori to work in partnership in the governance, planning, and delivery of health services [2].

Whanau Ora Commissioning Agency (WOCA) was the commissioning agency contracted by Te Puni Kōkiri/Ministry of Māori Development to deliver health and social services including COVID testing, COVID vaccination, and support for COVID positive Māori patients in the community. WOCA requested data from the Ministry of Health with the aim of increasing Māori vaccination rates by engaging with the unvaccinated. However, the Ministry of Health invoked several sections in the New Zealand Health Information Privacy Code, which places limits on the disclosure of health information. WOCA took legal action against the Ministry for refusing to release details of Māori who had not been vaccinated against COVID-19. It asked the Ministry to provide contact details of all unvaccinated Māori in the North Island of NZ including contact details, vaccination status and vaccination booking status of those who had not received any dose of the COVID-19 vaccine or have received only one dose of the vaccine.

The High Court ruled against the Ministry in a decision released on 1 November 2021, asking it to reconsider its decision to withhold the data [1]. The court noted the COVID-19 pandemic is a "once in a century public health crisis for Māori", for which there was readily available modelling data to show the impact for Māori in terms of the scale of deaths, hospitalisations and infections. The court found that the Ministry had been inconsistent in withholding data from WOCA that they had already shared with non-Māori organisations, and there are no concerns about the ability of WOCA to securely store, manage and delete the data. It ruled that the Ministry´s decision lacked objectivity, over-read the Health Information Privacy Code, was inconsistent with the principles of Te Tiriti o Waitangi, and also failed to understand that the mapping level option for WOCA to support a door-to-door approach was not an equally effective alternative.

[1] Te Pou Matakana Limited v Attorney-General NZHC 2942 [1 November 2021]
https://www.courtsofnz.govt.nz/assets/Uploads/2021-NZHC-2942.pdf

[2] Waitangi Tribunal. 2019. Hauora: Report on Stage One of the Health Services and Outcomes Kaupapa Inquiry. Wellington. Waitangi Tribunal. pp. 163–164S
